# Design, Synthesis and In Vitro Investigation of Novel Basic Celastrol Carboxamides as Bio-Inspired Leishmanicidal Agents Endowed with Inhibitory Activity against *Leishmania* Hsp90

**DOI:** 10.3390/biom11010056

**Published:** 2021-01-05

**Authors:** Ivan Bassanini, Silvia Parapini, Erica E. Ferrandi, Elena Gabriele, Nicoletta Basilico, Donatella Taramelli, Anna Sparatore

**Affiliations:** 1 Istituto di Scienze e Tecnologie Chimiche “Giulio Natta”—Consiglio Nazionale delle Ricerche, Via Mario Bianco 9, 20131 Milano, Italy; erica.ferrandi@scitec.cnr.it; 2 Dipartimento di Scienze Farmaceutiche, Università degli Studi di Milano, Via Mangiagalli 25, 20133 Milano, Italy; elena.gabriele88@gmail.com; 3 Dipartimento di Scienze Biomediche per la Salute, Università degli Studi di Milano, Via Mangiagalli 31, 20133 Milano, Italy; silvia.parapini@unimi.it; 4 Centro Interuniversitario di Ricerca sulla Malaria-Italian Malaria Network (CIRM-IMN), Università degli Studi di Milano, 20133 Milano, Italy; 5 Dipartimento di Scienze Biomediche, Chirurgiche e Odontoiatriche, Università degli Studi di Milano, Via Pascal 36, 20133 Milano, Italy; nicoletta.basilico@unimi.it; 6 Dipartimento di Scienze Farmacologiche e Biomolecolari, Università degli Studi di Milano Via Pascal, 36, 20133 Milano, Italy; donatella.taramelli@unimi.it

**Keywords:** celastrol, natural compounds, *Leishmania* Hsp90, Hsp90 inhibition, leishmanicidal compounds, protozoa

## Abstract

The natural triterpene celastrol (**CE**) is here used as lead compound for the design and synthesis of a panel of eleven **CE** carboxamides that were tested in vitro for their growth inhibitory activity against *Leishmania infantum* and *L.* *tropica* parasites. Among them, in vitro screening identified four basic **CE** carboxamides endowed with nanomolar leishmanicidal activity, against both the promastigotes and the intramacrophage *Leishmania* amastigotes forms. These compounds also showed low toxicity toward two human (HMEC-1 and THP-1) and one murine (BMDM) cell lines. Interestingly, the most selective **CE** analogue (compound **3**) was also endowed with the ability to inhibit the ATPase activity of the *Leishmania* protein chaperone Hsp90 as demonstrated by the in vitro assay conducted on a purified, full-length recombinant protein. Preliminary investigations by comparing it with the naturally occurring Hsp90 active site inhibitor Geldanamycin (**GA**) in two different in vitro experiments were performed. These promising results set the basis for a future biochemical investigation of the mode of interaction of celastrol and **CE**-inspired compounds with *Leishmania* Hsp90.

## 1. Introduction

**Figure 1:**
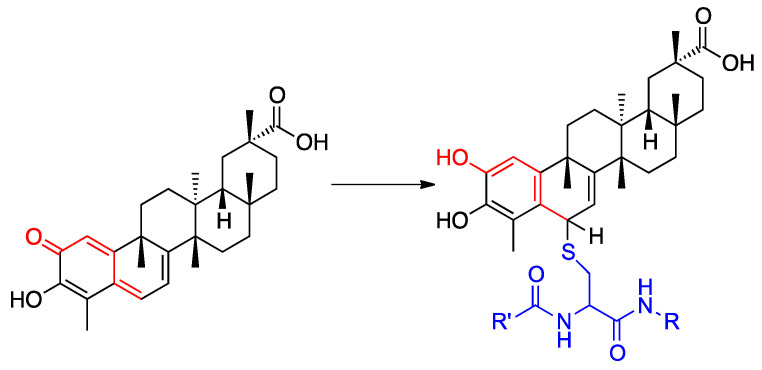
Structures of **CE** (**left**) and **CE**-protein covalent Michael adduct (**right**) formed on a cysteine residue.

Natural products have been used to date as a convenient source of potential lead compounds in the framework of drug discovery and development and they are now re-emerging as pivotal sources of chemical diversity in the post-genomics era [1]. Among them, plant-derived traditional remedies represent promising drug candidates due their potent pharmacological effects and generally encouraging toxicity profiles [2,3,4]. Celastrol (**CE**, Figure 1) is a naturally occurring biomolecule extracted from the traditional Chinese medicinal herb *Tripterygium wilfordii*, commonly referred to as *‘Thunder of God Vine’* [5,6]. Chemically, **CE** belongs to the class of natural quinone-methide triterpenes and possess a six-membered based pentacyclic structure which further classify it as a pentacyclic triterpenoid. Throughout the years the pharmacological profile of **CE** has been largely investigated highlighting its ability to modulate a variety of molecular targets both via the formation of covalent Michael adducts and/or by establishing non-covalent interactions (Figure 1) [7].

**CE** potentialities as drug have been reported for the treatment of different conditions including e.g., obesity, inflammatory, auto-immune and neurodegenerative diseases [2,8]. **CE** was also identified as potent antiproliferative agent for different human cancer cell lines interfering with different biological targets [2]. Among the others, **CE** can interact with the human variant of the 90kDa Heat Shock Protein (*h*Hsp90), a ubiquitous protein chaperone in Eukarya known to participate in crucial signaling pathways connected with cell growth and survival in stress-inducing conditions [9,10]. More recently, the growth inhibitory activity of **CE**-containing plant extracts or of the isolated natural compound against both *Plasmodium* and *Leishmania* parasites was reported [11,12].

The therapeutic options against leishmaniasis, a poverty-related protozoal disease, which causes 700,000 new cases worldwide with 26,000–65,000 deaths annually, are still limited and fraught with high costs, severe side effects and the rise and spread of drug resistance [13,14,15]. Interestingly, the transmission and the development of the various forms of this parasitosis are strictly related to the ability of the parasites of the genus *Leishmania* to differentiate into morphologically distinct life cycle stages via heat-shock induced events. Once transmitted from an infected phlebotomine sand-fly to a mammalian host, *Leishmania* parasites, in the form of flagellate promastigotes, are phagocytized by macrophages. In response to local factors including the rise of temperature, parasites differentiate at this point to the pathogenic, round and aflagellated amastigote stage. It was previously demonstrated that in these primitive eukaryotes the heat-shock induced differentiation stage is regulated, among others, by specific variants of Hsp90 [16,17].

**Figure 2:**
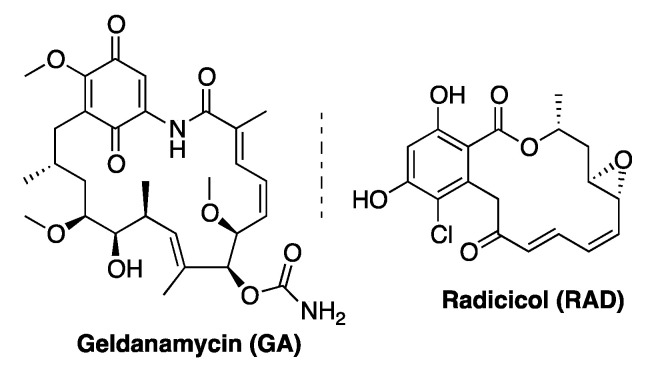
Structures of GA and RAD.

As a general strategy of target-oriented drug discovery, the pharmacological inhibition of Hsp90 chaperone cycle, which relies on ATP hydrolysis to assist the folding of a plethora of client proteins, has long been investigated by targeting its essential ATPase activity using ATP-competitive active inhibitors [18,19,20,21,22,23]. Accordingly, the two natural compounds radicicol (**RAD**) and geldanamycin (**GA**), classified as *h*Hsp90 active site inhibitors (Figure 2), were investigated for their growth inhibitory activity against *Leishmania* spp. promastigotes. The obtained results led to the possibility of exploiting (semi)synthetic analogues or specific formulations of **RAD** and **GA** as leishmanicidal agents targeting the *Leishmania* Hsp90 variants [24,25,26,27]. Recently, novel small molecules were identified as promising inhibitors of *Leishmania* spp. Hsp90 highlighting the potentialities of this approach in the research for novel antiprotozoal agents [28,29].

**Figure 3:**
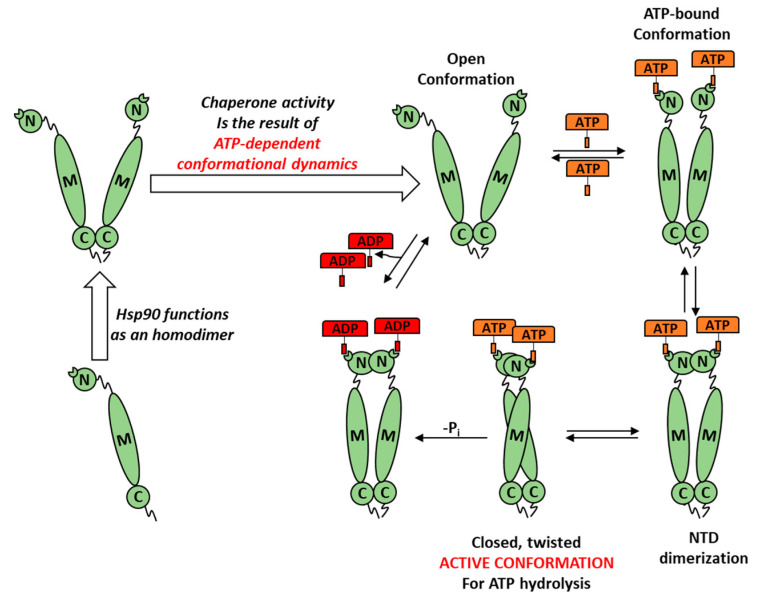
Schematic representation of the conformational dynamics characterizing a heat-shock chaperone. N = *N* terminus; C = *C* terminus and M = middle domain. ATP binding and its consequent hydrolysis modulates Hsp90 chaperone activity by regulating the transitions between conformational protein sub-states with distinct functional properties. The binding of allosteric ligands “selects” specific protein conformations via the modification of Hsp90 ATPase activity kinetics and the consequent interaction with client proteins [31].

In eukaryotic cells, the activity of Hsp90 is finely regulated by modulating its cytosolic expression and by the action of specific allosteric ligands. These are small proteins or peptides able to positively or negatively modulate the kinetics of Hsp90 ATP-dependent chaperone cycle by perturbing its complex conformational dynamics (Figure 3) [30].

Taking inspiration from this, the use of small molecules-based allosteric modulators of the Hsp90 chaperone cycle [31,32,33,34,35], has gained more and more attention in the field of innovative chemotherapies [36,37,38]. Unlike active site inhibitors, this class of compounds possesses in fact the unique ability to interfere with essential protein–protein interactions within the cells avoiding the insurgence of heat-shock responses, pro-survival mechanisms usually triggered by Hsp90 active site inhibitors [39,40]. Recently, detailed investigations revealed the ability of **CE** to allosterically block the formation of the protein-complex between human Hsp90 and the signal transduction protein Cdc37 resulting in a quite strong cytotoxicity and promising antiproliferative/anticancer activity [41,42,43,44,45,46]. Remarkably, basic **CE** derivatives have demonstrated a higher degree of selectivity of action in vitro when tested as potential antiproliferative agents interacting with the human Hsp90-Cdc37 system [47]. Kalayeh et al. also discovered a modest, yet promising, modulatory activity of **CE** against *Leishmania* Hsp90 chaperone cycle [17,29].

**Figure 4:**
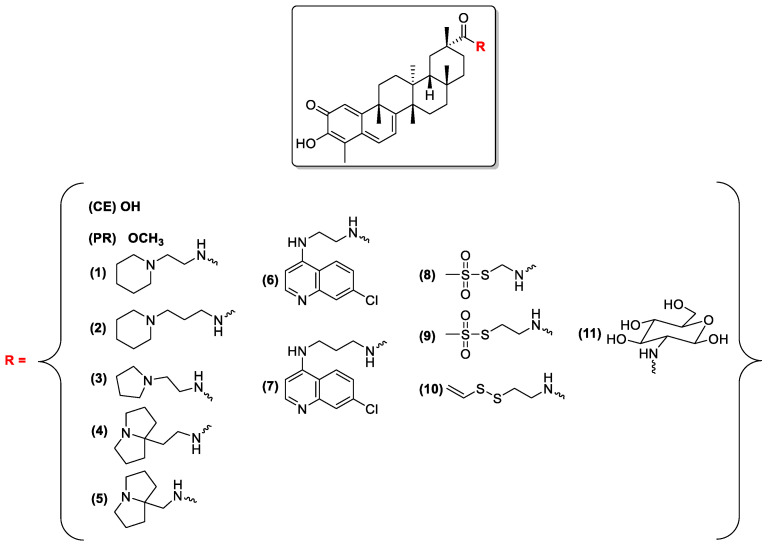
Structures of **CE**, **PR** and carboxamide derivatives **1**–**11**.

With the aim of developing novel leishmanicidal agents inspired from natural products, we designed and synthetized a small family of **CE** carboxamide derivatives (Figure 4). The compounds of Figure 4 are characterized by different polarities and lipophilicities thanks to the groups selected as amide decorations. Seven basic derivatives were prepared to assess the importance of the pH-sensitive nitrogen for antiprotozoal activity (compounds **1**–**7**). Among them, the piperidin-1-yl-ethanamine and the pirrolidin-1-yl-ethanamine **CE** carboxamides (compounds **1** and **2**) were previously reported as allosteric disruptors of the *h*Hsp90-Cdc37 protein complex but never tested against protozoa [47]. Moreover, a group of not basic CE carboxamides including three sulfur containing **CE** analogues (the two methanethiosulfonates **8** and **9** and the allyldisulfide **10**) and the more hydrophilic **CE**-glucosamide **11** were also synthesized and tested. Methanethiosulfonate and allyldisulfide derivatives have been designed because these moieties could react, as previously reported for other proteins [48,49,50], with the free thiol group of exposed cysteines in synergy with **CE** ability to form Michael adducts with electrophilic protein residues. Finally, the more lipophilic methyl ester derivative of **CE**, a natural compound named pristimerin (**PR**), was also included in our investigation.

All the mentioned compounds were screened in vitro for their growth inhibitory activity against cultures of *Leishmania* tropica and *L. infantum* promastigotes. Their cytotoxicity against normal cells was assessed using two human cell lines (the immortalized human microvascular endothelial line—HMEC-1—and the human monocytic line—THP-1) and a murine one (the bone marrow-derived macrophage—BMDM). The most promising compounds were also tested against intramacrophage cultures of *Leishmania* amastigotes and their selectivity of action was investigated, accordingly.

Intrigued by the improved performances as both Hsp90 inhibitors and antiproliferative agents demonstrated by **CE** basic carboxamides with the respect to the parental compound [47], the modulatory activity of the compounds of Figure 4 was also studied toward the homologous Hsp90 from *Leishmania braziliensis*. A preliminary in vitro comparison with **GA**, the known Hsp90 active site inhibitor endowed with leishmanicidal activity, was also conducted.

## 2. Materials and Method

### 2.1. Chemistry

#### 2.1.1. General Information

All commercially available solvents and reagents were used without further purification, unless otherwise stated. TLC was carried out on precoated 60 F254 plates (Merck, Darmstadt, Germany) using UV light. Flash column chromatography (flash column chromatography, CC) was performed using silica gel 60 (32–64 Mesh, Merck). Organic phases were dried over anhydrous sodium sulphate and evaporated under diminished pressure (1–2 kPa) at a bath temperature of 40 °C.

Synthetized compounds were characterized by means of ^1^H- and ^13^C-NMR spectra, melting point (Mp) and, with the exception of the previously reported compounds **1** and **3** [47], by high-resolution mass spectrometry. ^1^H- (300 MHz) and ^13^C-NMR (75 MHz) spectra were recorded on a Mercury 300VX spectrometer (Palo Alto, CA, USA). Peaks were assigned with 2D COSY experiments and are in agreement with the proposed structures; deuterated chloroform (CDCl_3_) was used as solvent. High-resolution mass spectra (HRMS) were conducted on a FT-Orbitrap mass spectrometer (ThermoFisher Scientific, Milan, Italy) in positive electrospray ionization (ESI) mode.

Purities of final compounds were determined by HPLC using CH_3_CN/H_2_O + CF_3_COOH gradient and a Purospher RP 18 5 μm column on an Elite Lachrom instrument (Hitachi, Chiyoda, Tokyo, Japan) equipped with a DAD detector; T_R_ = retention time. Compound characterization and HPLC data are reported in Appendix A.

#### 2.1.2. Preparation of Compounds **1**–**11**

Carboxamides were prepared as follow: under nitrogen atmosphere, HOBt (1 equiv.) and EDC-HCl (1.5 equiv.) were added to an ice-cooled solution of celastrol (1.0 equiv.) in dry DMF (170 mM) and stirred for 15 min. After that, TEA (1.5 equiv.) and 1.2 equiv. of the selected primary amine were added to reacting mixture which was allowed to warm to room temperature and stirred overnight. Target compounds were isolated after flash column chromatography on silica gel working with a gradient of MeOH in DCM.

### 2.2. Biological Assays

#### 2.2.1. Recombinant Expression of Full-Length *Leishmania braziliensis* Hsp90 (*Lb*Hsp90)

To evaluate in vitro the modulatory activity of CE, PR and CE caboxamides, a full-length recombinant Hsp90 from *L. braziliensis* (LbHsp90) was produced in *E. coli* BL21 DE3 harboring the pET28a_LbHsp90 coding for the C-terminal His-tagged target chaperone (genbank number*: XP_001567804.1*, full sequence is reported in Appendix B). Pratically, the plasmid pET28a_LbHsp90, kindly donated by professor J.C. Borges [51,52], was transformed in *E. coli* BL21 DE3 using the TransformAid Bacterial Transformation Kit (Thermo Fisher, Waltham, MA, USA). Subsequently, the obtained transformants were grown overnight in LB medium containing 30 μg mL^−1^ kanamycin (LB_kan30_) and then inoculated in 1 L SB _kan30_ medium (Yeast extract 30 g L^−1^, Tryptone 20 g L^−1^, NaCl 10 g L^−1^) at 37 °C and 220 rpm. When the OD_600_ reached 0.5–1, gene expression was induced by the addition of 2 mL of 1 M IPTG solution in water and the culture was shifted at 30 °C for 24 h. After cell harvesting by centrifugation (5000 rpm for 30 min), the cell pellet was resuspended in 20 mL of wash buffer (20 mM HEPES buffer, pH 7.0, 500 mM NaCl, 20 mM imidazole) and recombinant *E. coli* BL21DE3 cells were disrupted by ultrasonication. LbHsp90 was subsequently purified using a Nickel Sepharose 6 Fast Flow agarose resin (Ni-NTA) (GE Healthcare, Milan, Italy) as follows. Soluble protein fraction was separated from cell debris by centrifugation (10,000 rpm for 30min) after cell ultrasonication, and clear lysates were incubated with the Ni-NTA resin for 90 min at 4 °C under mild shaking and loaded onto a glass column (10 × 110 mm). The resin was then washed with 10mL of wash buffer and His-tagged *Lb*Hsp90 was eluted using a 3 step gradient (10 mL washing buffer containing 100, 200, and 300 mM imidazole, respectively) and dialyzed against 20 mM HEPES buffer (20 mM, pH 7.5), at 4 °C overnight. The protein content was measured using the Bio-Rad Protein Assay (Hercules, CA, USA) according to the Bradford method and the protein purity was verified by SDS-PAGE analysis (10% T, 2.6% C). It is worth noting that to avoid interferences with the assays described below, proteins were purified and conserved in HEPES buffer (20 mM, pH 7.5) in place of the standard PB-buffer. Reported data are the mean of two experiments run in duplicate.

#### 2.2.2. Determination of *Lb*Hsp90 ATPase Cycle Modulation In Vitro

Modulatory activity toward the ATPase cycle of *Lb*Hsp90 was determined using a Malachite Green Phosphate Assay Kit (Catalog Number MAK307, Sigma-Aldrich^®^, St. Louis, MO, USA). Analyses were run in a 96-well plate directly following the quantitative formation of a green complex between Malachite Green, molybdate, and free orthophosphate (P*i*) produced by ATP hydrolysis catalyzed by the chaperone. Color formation from the reaction was measured on a plate reader at 620 nm. Briefly, the IC_50_ of **CE** and its derivatives were extrapolated from a set of dose-response curves obtained by calculating the amount of P*i* produced by *Lb*Hsp90 in the presence of different compounds concentrations.

Reactions were run in HEPES buffer (20 mM, pH 7.5) containing 3 mM MgCl_2_ and 50 mM KCl in the presence of different compounds concentrations (12–500 mM), *Lb*Hsp90 (7 μM) and ATP (25 mM) at 27 °C for 60 min. OD_620_ was measured 30 min after Malachite Green reactants addition.

*Lb*Hsp90 native activity was measured by conducting the reaction in the presence of just *Lb*Hsp90 and ATP and extrapolating the concentration of P*i* obtained.

Three sets of control experiments, (1) ATP background hydrolysis, (2) compounds-related interferences and (3) protein-related background coloration, were run as follow:

(1) ATP (25 mM) incubated at 27 °C for 60 min
(2) Compounds (different concentrations) incubated with ATP (25 mM) at 27 °C for 60 min
(3) *Lb*Hsp90 (7 μM) at 27 °C for 60 min

#### 2.2.3. Evaluation of *Lb*Hsp90 ATPase Kinetics In Vitro

*Lb*Hsp90 (3.5 μM) was added to a solution prepared in HEPES buffer (20 mM, pH 7.5) containing KCl (100 mM), MgCl_2_ (1 mM), NADH (0.18 mM), L-lactate dehydrogenase (4 U mL^−1^), phosphoenolpyruvate (1 mM), pyruvate kinase (2.5 U mL^−1^) and the desired compound (dissolved in DMSO to a final concentration of 50 μM). The reaction was initiated by the addition of ATP (1 mM) and absorbance changes at 346 nm were monitored for 30 min at 30 °C.

Native *Lb*Hsp90 kinetics was measured as described but in the absence of DMSO. Two control experiments were run as follows:

(1) Efficiency of the enzymatic cascade by the used compounds: HEPES buffer (20 mM, pH 7.5) containing KCl (100 mM), MgCl_2_ (1 mM), ADP (1 mM), NADH (0.18 mM), L-lactate dehydrogenase (4 U mL^−1^), phosphoenolpyruvate (1 mM), pyruvate kinase (2.5 U mL^−1^);
(2) Interference of the tested compound(s) with the enzymatic cascade: HEPES buffer (20 mM, pH 7.5) containing KCl (100 mM), MgCl_2_ (1 mM), ADP (1 mM), NADH (0.18 mM), L-lactate dehydrogenase (4 U mL^−1^), phosphoenolpyruvate (1 mM), pyruvate kinase (2.5 U mL^−1^) and 50 μM compound.

Appendix B reports the detailed description of the enzymatic cascade used. 

#### 2.2.4. Promastigote Stage of Leishmania spp. Cultures and Antileishmanial Activity

Promastigote stage of *L. infantum* (MHOM/TN/80/IPT1, kindly provided by Dr. M. Gramiccia and Dr. T. Di Muccio, ISS, Roma) and *L. tropica* (MHOM/SY/2012/ISS3130) were cultured in RPMI 1640 medium (EuroClone) supplemented with 15% heat-inactivated fetal calf serum (EuroClone, Milan, Italy), 20 mM HEPES, and 2 mM L-glutamine at 23 °C. The MTT (3-[4.5-dimethylthiazol-2-yl]-2.5-diphenyltetrazolium bromide) method was used to estimate the 50% inhibitory concentration (IC_50_), with some modifications [53,54]. Compounds were dissolved in DMSO and then diluted with medium to achieve the required concentrations. Test compounds were placed in 96 wells round bottom microplates and seven serial dilutions made. Amphotericin B was used as the reference antileishmanial drug. Parasites were diluted in complete medium to 5 × 10^6^ parasites/mL and 100 μL of the suspension was seeded into the plates and incubated at 23 °C for 72 h. Then 20 μL of MTT solution (5 mg/mL) was added into each well, after 3 h the plates were centrifuged, the supernatants discarded and the resulting pellets dissolved in 100 μL of lysing buffer consisting of 20% (*w/v*) of a solution of SDS (Sigma), 40% of *N,N*-dimethylformamide (Merck) in H_2_O. The absorbance of the obtained solutions was measured at a wavelength of 550 nm with a reference at 650 nm with a microplate spectrophotometer (Synergy 4-Biotek, Milan, Italy) and analyzed with the Gen5^®^ software for data processing.

The results are expressed as IC_50_, the dose of compound that is necessary to inhibit cell growth by 50%; each IC_50_ value is the mean ± standard deviation of at least three separate experiments performed in duplicate.

#### 2.2.5. Intramacrophage Amastigotes Stage of *Leishmania* spp. Cultures and Antileishmanial Activity

For *Leishmania* infections, THP-1 cells were plated at 5 × 10^5^ cells/mL in 16-chamber Lab-Tek culture slides (Nunc, Frosinone, Italy) and treated with 0.1 μM phorbol myristate acetate (PMA, Sigma) for 48 h to achieve differentiation into macrophages. Cells were washed and infected with metacyclic *L. infantum* promastigotes at a macrophage/promastigote ratio of 1/10 for 24 h. Cell monolayers were then washed and incubated in the presence of test compounds for 72 h. Slides were fixed with methanol and stained with Giemsa. The percentage of infected macrophages in treated and non-treated cells was determined by light microscopy [55,56].

#### 2.2.6. Evaluation of Morphological Differentiation of *Leishmania* Cells

*L. infantum* promastigotes were diluted in complete medium to 4 × 10^7^ parasites/mL and 250 µL of the suspension were seeded into the 24 wells plates and incubated at 23 °C in the presence of geldanamycin, **CE** or compound 2 at the final concentration of 50 ng/mL. Cultures without drugs incubated at 23 °C or at 37 °C were used as negative and positive control, respectively. After 24 or 48 h of incubation, parasite morphology was evaluated by optical microscopy at 100× (Eclipse Ti Series, Nikon, Minato, Tokyo, Japan) on Giemsa stained smears (a mixture of eosin, methylene blue and Azure-B diluted 1:10). The percent of round promastigotes in treated and non-treated cells was determined. Images were taken at the same magnification by using a digital camera (Nikon Digital Sight). About 500 promastigotes were counted in 4 random fields.

#### 2.2.7. Cytotoxicity Assay on Different Cell Lines (HMEC-1, BMDM, THP1)

Cytotoxicity was evaluated on human microvascular endothelial cells (HMEC-1, provided by the Centers for Disease Control, Atlanta, GA, USA), on immortalized mouse C57Bl/6 bone marrow derived macrophages (BMDM) from wild type lineage (generated in the laboratories of Drs. Douglas Golenbock and Kate Fitzgerald, University of Massachusetts, Worcester, MA, USA) [57] and on a human acute monocytic leukaemia cell line (THP-1).

HMEC-1 were maintained in MCDB 131 medium (Invitrogen, Milan, Italy) supplemented with 10% fetal calf serum (HyClone), 10 ng/mL of epidermal growth factor, 1 μg/mL of hydrocortisone, 2 mM glutamine and 20 mM HEPES buffer, at 37 °C in 5% CO_2_. BMDM were maintained in DMEM (Euroclone) medium supplemented with 10% fetal bovine serum (HyClone, Celbio, Milan, Italy), 2 mM glutamine, and 25 mM of HEPES buffer solution (Euroclone). THP-1 were maintained in RPMI supplemented with 10% heat-inactivated FCS, 50 µM 2-mercaptoethanol, 10 µM sodium pyruvate, 20 mM HEPES, and 2 mM glutamine. In order to achieve differentiation into macrophages, cells were treated with 10 ng/mL phorbol myristate acetate (PMA) for 72 h.

For the cytotoxicity assay, cells were seeded in 96 well flat bottom tissue culture clusters (Costar, New York, NY, USA) at the following concentrations: HMEC 10^5^ cell/mL, BMDM at 10^6^ cell/mL, THP1 at 5 × 10^5^ cell/mL. After 24 h, cells were treated with serial dilutions of test compounds in a final volume of 200 µL/well. Cell proliferation was evaluated after 72 h using the MTT assay [53,54].

The absorbances were measured with a microplate spectrophotometer (Synergy 4-Biotek) at a test wavelength of 550 nm and a reference wavelength of 650 nm. The results are expressed as IC_50_ as the concentration inhibiting 50% of cell growth. Each IC_50_ value is the mean and standard deviation of at least three separate experiments performed in duplicate.

## 3. Results

### 3.1. Leishmanicidal Activity on Promastigotes Cultures and Cytotoxicity Evalutation

The leishmanicidal activity of **CE**, **PR** and the carboxamides **1**–**11** was first measured in vitro in two different species of *Leishmania* promastigotes (*L. infantum* and *L. tropica*) using amphotericin B (**AMP**) as reference drug. Since **CE** is reported to possess a plethora of different bioactivities [5,6,7], compounds’ aspecific cytotoxicity was estimated using different mammalian cell lines, both of human and murine origin. Specifically, the immortalized human microvascular endothelial cell line (HMEC-1) and two different macrophage cell lines (human monocytic THP-1 line and the bone marrow-derived murine BMDM line) were used. All the experiments were run as described in the M&M section [53,54]. The results are summarized in Table 1.

The lead compound **CE** exhibited a micromolar range activity against both *L. infantum* and *L. tropica* promastigotes and an almost equal aspecific cytotoxicity. The methyl ester **PR** showed similar or slightly improved leishmanicidal potency and cytotoxicity profile compared to **CE**.

In general, tertiary amine-based carboxamides (compounds **1**–**5**) were found to be as active or even more potent than **CE** itself, corroborating the consolidated hypothesis that a pH-responsive basic head improves the antiprotozoal activity [55,58]. Among them, the ethylene diamine-based piperidinyl and pyrrolidinyl analogues **1** and **3** were by far the most potent leishmanicidal agents with IC_50_ in the low-nanomolar range. Moreover, the cytotoxicity of this compounds was reduced against all the tested cell lines, with respect to **CE**. Interestingly, the propylene diamine-spaced piperidine derivative **2** was a less potent leishmanicidal compound than its inferior homologue **1**.

Both the pyrrolizidine derivatives **4** and **5** were more potent against *L. tropica* promastigotes than **CE** and generally less cytotoxic toward all the tested cell lines. Furthermore, compound **5**, the most potent of the two analogues against *Lb*Hsp90, was found to be also the least cytotoxic.

The quinoline derivatives **6** and **7** showed different potency and selectivity profiles. While the propylene diamine-based analogue **7** was generally comparable with **CE** in terms of potency against *L. infantum* promastigotes and cytotoxicity, the ethylene diamine-containing compound **6** outclassed the parent compound in terms of leishmanicidal activity.

The methanethiosulfonate derivatives **8** and **9** were modestly active toward both *Leishmania* spp. promastigotes (IC_50_ = 2–4 μM) and displayed negligible cytotoxicity mainly on BMDM cell line. The disulfide derivative **10** was found to be more potent than **CE** but of comparable cytotoxicity.

Finally, the glucosamine derivative **11** was totally inactive against all the tested cell lines (IC_50_ > 14 μM). This could be probably related to an insufficient cellular uptake due to its high polarity.

### 3.2. Leishmanicidal Activity on Amastigotes Cultures and Selectivity of Action

The four better performing compounds of Table 1 i.e., the **CE** derivatives **1**, **3**, **5** and **6**, whose nanomolar antileishmanial activity was combined with a reduced profile of aspecific cytotoxicity, were selected to be tested against intramacrophage amastigotes of *L. infantum*. Their selectivity of action was calculated with the respect of compounds’ IC_50_ on the human macrophage cell line THP-1 reported in Table 1. Results are summarized in Table 2.

All the tested compounds were found to be active against the amastigote stage of *L. infantum*, but with different degrees of selectivity. In detail, compounds **5** and **6**, bearing respectively a pyrrolizidine and an aromatic amine moiety showed a sub-micromolar antileishmanial activity but low selectivity. On the contrary, compounds **1** and **3**, demonstrated a leishmanicidal activity comparable with the reference drug **AMP** with higher selectivity when compared with compound **5** and **6**. Specifically, the ethylene diamine-spaced pyrrolidine derivative **3** exhibited the higher SI, equal to 21.

### 3.3. In Vitro Inhibition of LbHsp90

The in vitro performances of **CE**, **PR** and the carboxamides **1**–**11** (Figure 4) as potential inhibitors of the *Leishmania braziliensis* Hsp90 were investigated using Sigma-Aldrich^®^ Malachite Green Phosphate Assay Kit. The optimal concentrations of 7 μM *Lb*Hsp90 and ATP was 1 mM were used according to the protocol reported in the M&M section. The results expressed as IC_50_ (μM) are reported in Table 3.

Confirming previous reports [29], a modest yet detectable activity toward *Lb*Hsp90 was shown by **CE** which acted as a negative modulator of the chaperone ATPase activity.

The ethylenediamine-based basic derivatives (i.e., the piperidine, the pyrrolidine and the pyrrolizidine derivatives **1**, **3** and **5**) showed improved potency against *Lb*Hsp90 compared to the parent **CE**. Compound **5** was the most potent inhibitor of the series with ten-fold increase of activity compared to **CE** while the propylene diamine-based analogue (**2**) was the only basic, aliphatic carboxamide to be found inactive toward *Lb*Hsp90 up to 125 μM in the assay conditions. Although in compound **4** the two basic nitrogen atoms are separated by three carbon atoms, this compound is still very potent, probably because one carbon is included in the bicyclic moiety making the alkyl chain less flexible than the propylene one.

The quinoline derivatives **6** and **7**, inspired by the aromatic portion of the antiprotozoal drug chloroquine, presented the same pattern described for the basic derivatives **1**–**5**. In fact, the ethylene diamine-based analogue **6** worked as an inhibitor of *Lb*Hsp90 with a potency comparable with that of the carboxamides **1**, **3**–**5**. On the contrary, in analogy to compound **2**, the propylenediamine-containing derivative **7** was inactive up to 125 μM.

The chaperone ATPase modulatory activity of the two methanethiosulfonates analogues **8** and **9** was again found to be sensitive to the length of the alkyl chain spacer linking the common **CE** core and the methanethiosulfonate moiety, with the shorter compound **8** inhibiting *Lb*Hsp90 with IC_50_ = 67 μM in the assay conditions, while the longer one **9** was inactive up to a concentration of 125 μM.

Interestingly, the allyldisulfide ethanamide derivative **10**, devoid of any coordinating polar or pH-responsive groups in the carboxamide side chain, did not show any relevant modulatory activity up to a concentration of 125 μM. The glucosamide derivative **11** inhibited *Lb*Hps90 ATPase cycle with an IC_50_ of 47 μM, similarly to other glycosides previously reported as allosteric modulators of the human Hsp90 [33].

Sustaining the hypothesis that hydrogen bonding groups could promote a tighter interaction with *Lb*Hsp90 and **CE**-based scaffolds, Pristimerin (**PR**), which lacks any H-bonding promoting residues, resulted inactive on the protein (IC_50_ > 500 μM).

### 3.4. ATP-Competitve vs. Non-Competitive Hsp90 Modulation

**CE** and the **CE**-carboxamides **1** and **3** were previously reported in the literature as compounds endowed with antiproliferative activity in virtue of their ability to allosterically disrupt the protein complex between the human Hsp90 and the protein Cdc37 [41,42,43,44,45,46,47]. By definition, allosteric modulation is a type of non-competitive inhibition/activation of a target protein which is sometimes translated in a detectable variation of an enzymatic activity, like in the case of the ATPase activity of Hsp90 chaperones. To preliminarily investigate the nature of the interaction between this family of compounds and *Leishmania* Hsp90, the ATPase inhibitory activity of the known active site inhibitor, ATP-competitive **GA** toward *Lb*Hp90 was compared with that of **CE** and the basic derivative **3**.

Accordingly, an in vitro assay developed for the evaluation of non-competitive allosteric modulators, which exploits ATPase kinetics data, was used [31,33]. Briefly, the decrease in absorbance deriving from the continuous oxidation of NADH to NAD^+^ produced by a multi-enzymatic cascade triggered by *Lb*Hp90-catalyzed ATP hydrolysis is followed spectrophotometrically over the course of 15 min (for details see Appendix B). All the experiments were normalized using the native ATPase activity of *Lb*Hsp90 measured in the presence of DMSO, the co-solvent selected for compounds solubilization. According to literature procedures, the compounds were incubated at the threshold concentration of 50 μM in the presence of 3.5 μM *Lb*Hsp90 and 1 mM ATP [31,33].

**Figure 5:**
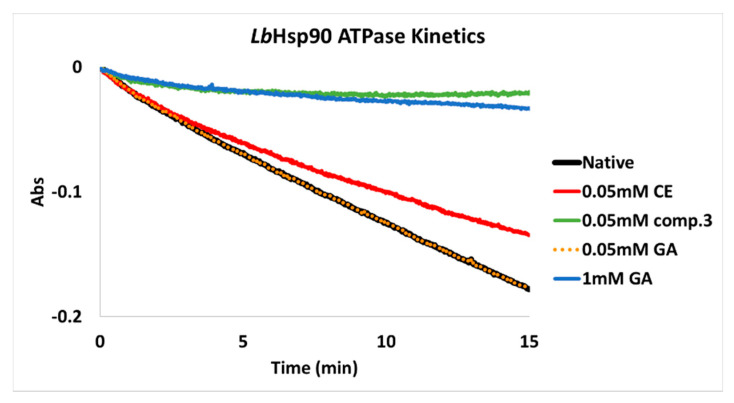
*Lb*Hsp90 ATPase kinetics: native (black); in the presence of 0.05 mM **CE** (red), compound **3** (green) and **GA** (orange) and of 1 mM **GA** (blue).

The *Lb*Hsp90 ATPase kinetics modulated by **CE** and compound **3** (red and green lines, respectively) are reported and compared with the native chaperone cycle of the protein (black line) in Figure 5. As it was extrapolated from the reported curves (Appendix B), **CE** reduced the native *Lb*Hsp90 ATPase activity of 30% while the carboxamide **3** inhibited 95% of it in the presence of 20-fold excess of ATP. These results are in agreement with the higher potency shown by compound **3** on the protein in comparison with **CE** (lower IC_50_ in Table 3).

Since **GA** acts on Hsp90 chaperone targeting their active site via a ATP-competitive mechanism, its ATPase inhibitory activity is, by definition, strictly dependent on ATP concentration. Accordingly, in the assay conditions (1 mM ATP) and when **GA** is used at the same concentration of **CE** and compound **3** (50 μM), its inhibitory potency toward *Lb*Hsp90 was negligible (Figure 5, orange line). Complete inhibition was instead achieved only conducting the assay in the presence of 1 mM **GA** (Figure 5, blue line). **GA**- inhibition of *Lb*Hsp90 ATPase kinetics at crescent concentrations–from 10 μM to 1 mM–are reported in Appendix B.

The treatment of the elongated and flagellate promastigotes of *L. donovani* with sub-IC_50_ concentrations of the Hsp90 active site inhibitor **GA** was reported to induce morphological differentiation into a round, amastigote-like stage. This morphological differentiation was also coupled with the expression of amastigote-specific proteins. Moreover, upon long-term exposures to **GA**, the treated promastigotes activated a series of cellular pro-survival mechanisms, defined as heat-shock responses, which restored and/or made up for Hsp90 chaperon functions resulting in an embryonal drug resistance to **GA** [16,27]. At variance to **GA** and Hsp90 active site inhibitors in general, allosteric modulators could be able to interfere with Hsp90-mediated folding events and protein–protein interactions while avoiding the insurgence of heat-shock responses [39,40].

**Figure 6:**
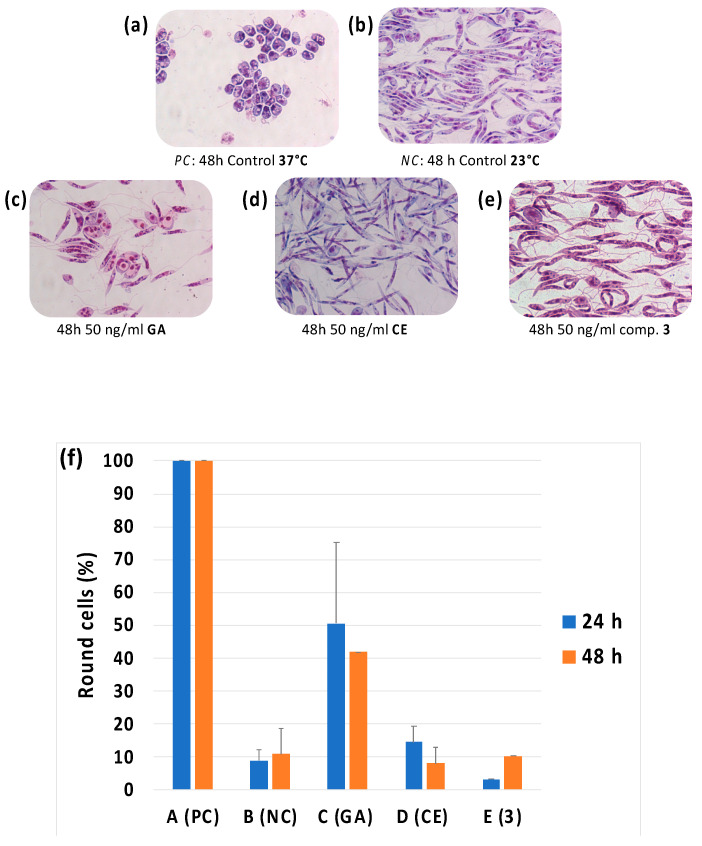
Optical microscope photography (magnification ×1000) of Giemsa-stained smears of *L. infantum* promastigotes incubated for 48 h (**a**) at 37 °C to induce a reversible differentiation, positive control (*PC*); (**b**) at 23 °C, negative control (*NC*); (**c**) at 23 °C in the presence of 50 ng/mL of **GA**, (**d**) **CE** and (**e**) compound **3**. In (**f**) the percentage of round amastigote-like cells after 24 h and 48 h of incubation is reported in the different conditions.

Aiming at collecting additional, yet qualitative, preliminary data comparing **GA** to the potential non-ATP-competitive Hsp90 inhibitors, **CE** and compound **3**, their morphological effects of the exposure of *Leishmania* promastigotes were evaluated *in vitro*. The compounds were incubated (23 °C, physiological pH) with *L. infantum* promastigotes at a sub-IC_50_ concentration of 50 ng/mL. IC_50_ of **GA** on *L. infantum* (0.14 ± 0.04 μM) promastigotes was measured as described in the M&M section. After 24 h and 48 h the presence of round, amastigote-like cells, was monitored (Figure 6). A negative and positive control experiment (*NC* and *PC*, Figure 6a,b) were also conducted growing the promastigotes at 23 °C and 37 °C, respectively.

## 4. Discussion

In the quest for novel antiprotozoal agents, the identification of novel targets and mechanisms of actions is highly cherished to surpass the insurgence and spread of drug resistant parasite strains. Not to be forgotten, the cytotoxicity and the lack of selectivity of action of modern and classical leishmanicidal agents is well-known and represents a harming issue of public health [13,14,15,55,56,58].

With the aim of developing novel antileishmanial agents exploiting the rich source of chemical diversity represented by bioactive natural products, a small family of carboxamide derivatives of the natural and bioactive triterpene **CE** (Figure 4) was designed, synthesized and screened in vitro.

The tested compounds were synthetized applying HOBt/EDC coupling chemistry to be characterized by different polarities and lipophilicities selecting diverse groups as amide decorations: cyclic tertiary amines, 4-aminoquinolines, methanethiosulfonates, an allyldisulfide and a glucosamine.

In vitro investigations were at first focused on the definition of compounds’ aspecific cytotoxicity and leishmanicidal activity against *Leishmania* promastigotes. Accordingly, the growth inhibitory activity of **CE** carboxamides, **PR** and of the parental compound **CE** were measured against *L. infantum* and *L. tropica* promastigotes. Cytotoxicity was estimated using the human HMEC-1 and THP-1 cell lines and the murine macrophage line BMDM (Table 1).

As described in the Results section, the best performing **CE** carboxamides (compounds **1**, **3**, **5** and **6**) were all characterized by the presence of a basic head as substituent of the amide side chain and all displayed reduced cytotoxicity and improved leishmanicidal potency with the respect to **CE** itself.

Among them, compound **3** showed a low-nanomolar activity (IC_50_ < 0.1 μM) against both *L. infantum* and *L. tropica* promastigotes and the lowest cytotoxicity. The methanethiosulfonates **8** and **9** and the glucosamine derivative **11** were found to be poorly active against all the tested cell lines probably due to issues related to cellular uptake.

The most active toward *Leishmania* promastigotes and least cytotoxic carboxamides (i.e., the mentioned compounds **1**, **3**, **5** and **6**) were also tested in vitro against intramacrophage amastigotes of *L. infantum* (Table 2). While compounds **5** and **6**, bearing respectively a pyrrolizidine and an aromatic amine moiety showed a sub-micromolar antileishmanial activity but low selectivity, compounds **1** and **3** demonstrated higher leishmanicidal activity and selectivity. Specifically, the ethylene diamine-spaced pyrrolidine **CE**-derivative **3** showed a growth inhibitory activity comparable with the reference drug **AMP** and a SI equal to 21.

**CE** and **CE**-related compounds are generally described in the literature as promising candidates for the development of novel drugs targeting different human pathologies in virtue of the different biological activities that have been correlated to the administration of **CE** itself or **CE**-enriched plant extracts [2,5,6,7,8,11,12,29]. From a mechanistic point of view, these bioactivities can be ascribed to an aspecific cytotoxicity deriving from a complex of possible and simultaneously operating mechanisms of action potentially involving different protein targets via reversible or covalent binding [7]. As previously described, **CE** was reported to be endowed with the ability to allosterically disrupt the protein complex between the human Hsp90 and the protein Cdc37 [41,42,43,44,45,46,47]. Moreover, in a following study, **CE** basic carboxamides (including compounds **1** and **3**) have been described as disruptors of the Hsp90-Cdc37 protein complex characterized by an improved antiproliferative action and selectivity against tumor cells [47].

On this basis, to preliminary investigate if Hsp90 inhibition could be involved in their promising described leishmanicidal activity, the modulatory activity of **CE** itself and of the basic **CE** carboxamides of Figure 4 was screened toward the ATPase activity of a homologous Hsp90 from *Leishmania braziliensis*. As a general consideration, the IC_50_ values reported in Table 3 can be considered as an estimation of the modulatory effect on *Lb*Hsp90 ATPase cycle of the tested compounds to facilitate the comparison of their inhibitory potency in the assay conditions applied. The Hsp90 chaperone on which the tests were run is in fact an isolated and purified recombinant protein which is devoid of its natural cellular environment and must be used in a concentration sufficiently high to fulfill the sensibility of the assay. According to its native ATPase activity, 7 μM *Lb*Hsp90 was the optimum concentration selected, based on the limit of quantification of the Sigma-Aldrich^®^ Malachite Green Phosphate Assay Kit.When incubated in the presence of *Lb*Hps90 (Table 3), the **CE** carboxamides either behaved as inhibitors of the chaperone ATPase activity (compounds **1**, **3**–**6**, **8** and **11**) or they were found to be basically inactive up concentrations of 125–150 μM (compounds **2**, **7**, **9** and **10**). Given their different structural features and the respective IC_50_ on the target proteins, some qualitative speculations can be made about the requirements for the interaction of this class of **CE**-derivatives with a still-to-be characterized allosteric pocket *Lb*Hsp90. It can be argued in fact that steric hindrance and the presence of a H-bond acceptor on the amide side chain play an important role in the interaction with the protein. Accordingly, compounds **1**, **6**, and **8** were all active against *Lb*Hsp90 while their homologues **2**, **7** and **9** (containing just one methylene more) did not show any modulatory activity on the chaperone regardless the analogue nature of their amide side chains. Moreover, the methyl ester derivative **PR** (devoid of any N/NH or Lewis basic group) and the lipophilic disulfide **10** were inactive up to 500 and 125 μM, respectively.

As previously stated, the use of ATP-competitive active site inhibitors of Hsp90 as potential antiprotozoal agents is well documented in the literature [11,16,25,26]; among them the natural antibiotics **GA**, known for its high cytotoxicity [59,60], was largely investigated.

Interestingly, upon long-term exposures to **GA**, the rise of drug resistance, in the form of heat-shock responses, was reported for treated cultures of *Leishmania* promastigotes. Usually, these pro-survival mechanisms are evident, besides in the pattern of expressed signaling proteins, also at a morphological level, since promastigotes are prone to differentiate into a round shaped amastigote-like state [16,27]. Generally speaking, these effects are usually voided by the use of non-ATP-competitive modulators of the ATPase cycle of Hsp90 chaperones [39,40]. To preliminary investigate whatever the ATPase inhibitory activity of **CE** and **CE** carboxamides could be or not ATP-competitive in nature, a comparison between the inhibitory activity toward *Lb*Hsp90 ATPase cycle of **CE** and the basic carboxamide **3,** a previously described allosteric disruptor the complex Hps90-Cdc37 in humans [47], was conducted in vitro operating ad hoc designed kinetics assays.

When **GA** (50 μM) was tested for its ability to modulate *Lb*Hsp90 ATPase kinetics in the presence of an excess of ATP (1 mM), no relevant effects were identified (Figure 5, yellow line). According to its ATP-competitive mechanism of chaperone inhibition, when 1 mM GA was used a *Lb*Hsp90 ATPase activity was completely inhibited (Figure 5, blue line). In contrast, **CE** and carboxamide **3** exhibited a strong negative modulatory effect toward the kinetics of the chaperone (30% and 95% of inhibition, respectively; Figure 5 green and red lines). These preliminary results could highly suggest that for **CE** and **CE**-carboxamides, as it was reported for compound **3** [47], a non-ATP-competitive mode of interaction with *Leishmania* Hsp90 could be operating.

Moreover, the performances of **GA**, **CE** and compound **3** were compared in an in vitro experiment designed to assess their effects on the cell morphology of cultures of *Leishmania* promastigotes treated with sub-IC_50_ concentrations of the listed compounds (Figure 6). *Leishmania* promastigotes, elongated and flagellate cells, tend in fact to morphologically differentiate into round amastigote-like cells when treated with ATP-competitive Hsp90 active site inhibitors, whose action on the protein “mimics” the heat-shock events and the responses which trigger lifecycle stage progression in parasites [16,27]. Accordingly, **GA** triggered a morphological differentiation on *Leishmania* promastigotes which turned into round amastigote-like cells in *ca* 40% of the total amount of analyzed cells (Figure 6c). On the contrary, neither **CE** nor compound **3** produced this effect on the treated parasites (Figure 6c,d) which remained in their promastigote state, qualitatively and preliminary sustaining the hypothesis of a non-ATP-competitive mode of interaction with the chaperone, in line with **CE** well-characterized mechanism of inhibition of the human Hsp90 homologue [41,42,43,44,45,46,47].

## 5. Conclusions

In this work, the natural and bioactive triterpene celastrol was used as lead compound for the design and synthesis of bioinspired, novel potential leishmanicidal agents. Four compounds, the basic **CE**-carboxamides **1**, **3**, **5** and **6** were found to be endowed with a strong growth inhibitory effect on *Leishmania tropica* and *L. infantum* promastigotes joined with a reduced aspecific cytotoxicity with the respect of the parental compound **CE**. Among them, the basic compound **3** showed also a promising nanomolar activity against intramacrophage *Leishmania* amastigotes together with a good selectivity against the THP-1 human cell line. These features address compound **3** as good candidate for a detailed SAR investigation to develop novel bio-inspired leishmanicidal agents further optimizing the molecular skeleton of **CE**.

Moreover, the potential modulatory effects of **CE** and its derivatives toward the ATPase cycle of the *Leishmania* chaperone Hsp90 was investigated. Depending on their different structural features, the novel **CE**-analogues were generally more potent than **CE** itself in inhibiting the ATPase activity of a recombinantly expressed Hps90 from *L. braziliensis.* Interestingly, the four basic carboxamides **1**, **3**, **5** and **6**, the most potent, least toxic and promising leishmanicidal agents of this series, were also found to be the most active compounds on the chaperone and potentially acting through a non-ATP-competitive mode of action.

Based on these preliminary, yet promising, in vitro data, the role of the ATPase cycle modulation of *Leishmania* Hsp90 in the leishmanicidal activity of **CE**-carboxamides (especially **1** and **3**) will be deeply investigated to elucidate whether this chaperone could be a biological target of these compound in vivo. In parallel, the biochemical features of their interactions with the protein at a molecular level will be investigated in ad hoc designed in vitro experiments and the results will be reported in a future paper.

## Figures and Tables

**Table 1:** Antileishmanial activity on promastigotes of *L. infantum* or *L. tropica* and cytotoxicity on human and murine cell lines.

*Leishmania* Promastigotes (IC_50_, μM)			Healthy Cell Lines (IC_50_, μM)		
	*L. infantum*	*L. tropica*	HMEC-1	THP-1	BMDM
**CE**	0.21 ± 0.07	0.49 ± 0.17	0.38 ± 0.08	0.90 ± 0.30	1.76 ± 0.43
**PR**	0.26 ± 0.02	0.24 ± 0.04	N.T. ^1^	1.10 ± 0.35	1.27 ± 0.09
**1**	0.06 ± 0.02	0.09 ± 0.03	0.51 ± 0.08	2.08 ± 0.24	3.61 ± 0.38
**2**	0.26 ± 0.06	0.21 ± 0.04	N.T. ^1^	1.78 ± 0.48	N.T. ^1^
**3**	0.06 ± 0.01	0.08 ± 0.04	0.53 ± 0.17	2.79 ± 0.44	4.53 ± 0.05
**4**	0.25 ± 0.08	0.20 ± 0.04	0.35 ± 0.16	1.89 ± 0.43	2.59 ± 0.71
**5**	0.12 ± 0.02	0.12 ± 0.05	0.12 ± 0.03	1.88 ± 0.34	2.68 ± 0.81
**6**	0.09 ± 0.03	0.14 ± 0.03	0.40 ± 0.03	0.85 ± 0.24	2.15 ± 0.02
**7**	0.20 ± 0.01	0.15 ± 0.04	0.13 ± 0.01	0.48 ± 0.17	1.78 ± 0.53
**8**	4.05 ± 1.96	3.44 ± 1.12	3.03 ± 0.01	2.51 ± 0.87	13.88 ± 2.78
**9**	2.31 ± 0.28	3.07 ± 1.36	4.07 ± 0.05	3.28 ± 0.90	12.96 ± 2.48
**10**	0.46 ± 0.12	0.34 ± 0.21	0.17 ± 0.04	0.93 ± 0.27	2.86 ± 0.17
**11**	14.45 ± 1.96	15.15 ± 2.71	>14.17	>15.90	>15.90
**AMP**	0.16 ± 0.05	0.17 ± 0.02	>20	>20	>20

Reported data are the mean of three experiments run in duplicate. Amphotericin B was used as reference drug.; ^1^ N.T.: not tested.

**Table 2:** Antileishmanial activity on intramacrophage amastigotes of *L. infantum* and evaluation of selectivity toward THP-1 cultures.

	*L. infantum* Amastigotes IC_50_ (μM) ^1^	S.I. ^2^
**1**	0.19 ± 0.11	11
**3**	0.13 ± 0.09	21
**5**	0.52 ± 0.28	3.6
**6**	0.66 ± 0.06	1.3
**AMP**	0.18 ± 0.03	>109

^1^ Reported data are the mean of three experiments. Amphotericin B was used as reference drug; ^2^ Selectivity index: ratio between compounds’ IC_50_s on THP-1 cell line (Table 1) and toward *L. infantum* amastigotes.

**Table 3:** In vitro inhibition of the ATPase activity of *Lb*Hsp90.

Compound	IC_50_ (μM) ^1^
**CE**	201 ± 10
**1**	83 ± 2
**2**	>125
**3**	81 ± 1
**4**	48 ± 3
**5**	20 ± 2
**6**	65 ± 4
**7**	>125
**8**	67 ± 3
**9**	>150
**10**	>125
**11**	47 ± 5
**PR**	>500

^1^ The reported data are the mean of two experiments run in triplicates.

**Figure A1:**
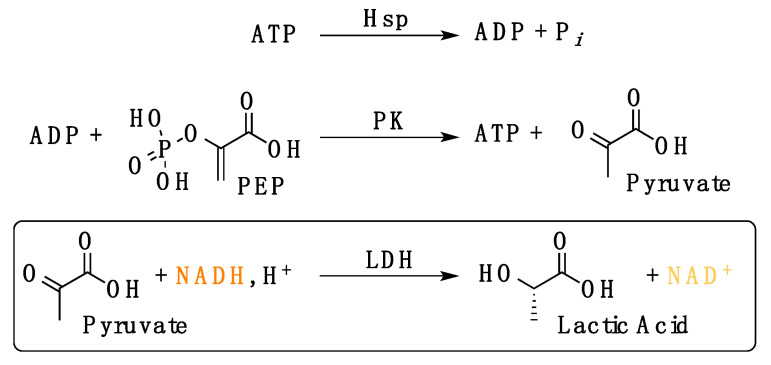
Enzymatic cascade reactions to spectroscopically evaluate *L.* Hsp90 ATPase kinetics. The ADP produced by ATP hydrolysis during the chaperone cycle reacts with PEP (phosphoenolpyruvate) to form pyruvate in a reaction catalyzed by the enzyme pyruvate kinase (PK), whereby ATP is regenerated. Pyruvate then continues the cascade process and is reduced to lactic acid by action of lactate dehydrogenase (LDH), whereby NADH is oxidized to NAD^+^.

## Data Availability

Not applicable.

## Abbreviations

DMF	dimethylformamide
DCM	dichloromethane
HOBt	hydroxybenzotriazole
EDC	1-ethyl-3-(3-dimethylaminopropyl)carbodiimide
TEA	triethylamine
s	singlet
bs	broad singlet
d	doublet
bd	broad doublet
t	triplet.

## Appendix A

In this appendix details of the compound characterizations and HPLC methods are reported.

*(2R,4aS,6aS,12bR,14aS,14bR)-10-hydroxy-2,4a,6a,9,12b,14a-hexamethyl-11-oxo-N-(2-(piperidin-1-yl)ethyl)-1,2,3,4,4a,5,6,6a,11,12b,13,14,14a,14b-tetradecahydropicene-2-carboxamide* (**1**) [47]. Yield: 64%; orange solid. Mp: 137.0–138.6 °C. ^1^H-NMR (CDCl_3_): δ 7.01 (d, *J* = 7.1 Hz, 1H), 6.77 (s, 1H, amide NH), 6.51 (s, 1H), 6.34 (d, *J* = 7.2 Hz, 1H), 3.25 (q, *J* = 5.3 Hz), 2.66–2.62 (m, 7H), 2.44 (bd, 1H), 2.21 (s, 3H), 2.17–2.04 (m, 4H), 1.97–1.84 (m, *J* = 14.6, 4.9 Hz, 8H), 1.70–1.47 (m, 8H), 1.47–1.44 (m, 4H), 1.26 (s, 5H), 1.16 (s, 3H), 1.11 (s, 3H), 1.11–1.10 (s, 1H), 0.90–0.83 (m, 1H), 0.63 (s, 3H). ^13^C-NMR (CDCl_3_): δ 178.3, 178.0, 170.3, 164.8, 146.0, 134.0, 127.3, 119.5, 118.0, 117.1, 54.4, 53.8, 45.1, 44.4, 43.0, 40.2, 39.4, 38.1, 37.5, 36.4, 34.9, 33.7, 33.5, 31.6, 31.2, 30.8, 30.0, 29.4, 28.7, 23.5, 21.8, 18.2, 10.2. HPLC purity: >99%.

*(2R,4aS,6aS,12bR,14aS,14bR)-10-hydroxy-2,4a,6a,9,12b,14a-hexamethyl-11-oxo-N-(3-(piperidin-1-yl)propyl)-1,2,3,4,4a,5,6,6a,11,12b,13,14,14a,14b-tetradecahydropicene-2-carboxamide* (**2**). Yield: 46%; orange solid. ^1^H-NMR (CDCl_3_): δ 7.02 (d, *J* = 7.1 Hz, 1H), 6.78 (s, 1H), 6.49 (s, 1H), 6.33 (d, *J* = 7.2 Hz, 1H), 3.21 (q, *J* = 5.3 Hz, 2H), 2.66–2.62 (m, 7H), 2.44 (bd, 1H), 2.21 (s, 3H), 2.17–2.04 (m, 4H), 1.97–1.84 (m, *J* = 14.6, 4.9 Hz, 8H), 1.70–1.45 (m, 10H), 1.47–1.44 (m, 4H), 1.25 (s, 5H), 1.14 (s, 3H), 1.10 (s, 3H), 1.11–1.10 (s, 1H), 0.90–0.83 (m, 1H), 0.63 (s, 3H). ^13^C-NMR (CDCl_3_): δ 170.5, 164.8, 146.0, 134.2, 127.3, 119.5, 118.0, 117.1, 58.4, 54.6, 45.1, 44.5, 43.0, 40.01, 39.96, 39.4, 38.1, 36.4, 35.0, 33.8, 33.6, 31.59, 31.48, 30.8, 29.9, 29.66, 29.61, 29.43, 29.34, 28.7, 25.2, 23.80, 23.68, 21.7, 18.2, 10.2. HRMS (ESI) *m/z* calcd for C_37_H_55_ N_2_O_3_ [M + H]^+^: 575.4213, found: 587.4218. 575.4216. HPLC purity: >98.7%.

*(2R,4aS,6aS,12bR,14aS,14bR)-10-hydroxy-2,4a,6a,9,12b,14a-hexamethyl-11-oxo-N-(2-(pyrrolidin-1-yl)ethyl)-1,2,3,4,4a,5,6,6a,11,12b,13,14,14a,14b-tetradecahydropicene-2-carboxamide* (**3**) [47]. Yield: 46%; orange solid. Mp: 135.6–138.0 °C. ^1^H-NMR (CDCl_3_): δ 7.01 (d, *J* = 7.0 Hz, 1H), 6.80 (s, 1H), 6.51 (s, 1H), 6.34 (d, *J* = 7.1 Hz, 1H), 3.26 (q, *J* = 5.2 Hz, 2H), 2.64 (bs, 6H), 2.44 (d, *J* = 15.6 Hz, 1H), 2.21–2.04 (m, 6H), 2.01–1.98 (m, 7H), 1.70–1.44 (m, 11H), 1.25 (s, 5H), 1.16 (s, 3H), 1.11 (s, 3H), 1.20–0.96 (m, 1H), 0.91–0.83 (m, 1H), 0.63 (s, 3H). ^13^C-NMR (CDCl_3_): δ 178.3, 178.0, 170.3, 164.7, 146.0, 134.0, 127.3, 119.5, 118.0, 117.0, 54.4, 53.8, 45.1, 44.4, 43.0, 40.2, 39.4, 38.1, 37.4, 36.4, 34.9, 33.7, 33.5, 31.6, 31.2, 30.8, 30.0, 29.4, 28.7, 23.5, 21.7, 18.2, 10.2. HPLC purity: >99%.

*(2R,4aS,6aS,12bR,14aS,14bR)-N-(2-(hexahydro-1H-pyrrolizin-7a-yl)ethyl)-10-hydroxy-2,4a,6a,9,12b,14a-hexamethyl-11-oxo-1,2,3,4,4a,5,6,6a,11,12b,13,14,14a,14b-tetradecahydropicene-2-carboxamide* (**4**). Yield: 69%; orange solid. Mp: 235.7–237.0 °C. ^1^H-NMR (DMSO-*d*_6_): δ 8.68 (s, 1H), 8.34 (s, 1H), 7.05 (d, *J* = 6.6 Hz), 6.37 (s, 1H), 6.33 (d, 1H, *J* = 7.2 Hz), 2.99 (bs, 2H), 2.82 (bs, 2H), 2.32–2.15 (m, 4H) 2.07 (s, 3H), 1.96–1.92 (m, 2H), 1.79–1.46 (m, 20H), 1.36 (s, 3H), 1.20 (s, 3H), 1.05 (s, 3H), 0.99 (s, 3H), 0.89–0.85 (m, 2H), 0.53 (s, 3H). ^13^C-NMR (DMSO-*d*_6_): δ 178.3, 177.3, 170.5, 164.7, 146.0, 134.1, 127.3, 119.5, 117.9, 117.0, 73.4, 72.6, 55.3, 55.1, 49.4, 45.1, 44.4, 43.0, 39.9, 39.3, 38.2, 37.4, 36.8, 36.5, 35.0, 33.7, 33.5, 31.6, 31.2, 30.8, 30.0, 29.3, 28.7, 27.0, 24.9, 21.7, 18.1, 10.2. HRMS (ESI) *m/z* calcd for C_38_H_55_N_2_O_3_ [M + H]^+^: 587.4212, found: 587.4218. HPLC purity: 98.1%.

*(2R,4aS,6aS,12bR,14aS,14bR)-N-((hexahydro-1H-pyrrolizin-7a-yl)methyl)-10-hydroxy-2,4a,6a,9,12b,14a-hexamethyl-11-oxo-1,2,3,4,4a,5,6,6a,11,12b,13,14,14a,14b-tetradecahydropicene-2-carboxamide* (**5**). Yield: 50%; orange solid. Mp: 250.8–254.7 °C. ^1^H-NMR (DMSO-*d*_6_): δ 8.65 (s, 1H), 8.34 (d, *J* = 7.2 Hz, 1H), 8.22 (d, *J* = 8.7 Hz, 1H), 8.02 (bs, 1H), 7.83 (s, 1H), 7.54 (d, *J* = 8.7 Hz, 1H), 6.96 (d, *J* = 5.7 Hz, 1H), 6.60 (d, *J* = 7.2 Hz, 1H), 6.26 (s, 1H), 6.13 (d, *J* = 5.7 Hz, 1H), 3.36–3.25 (m, 4H), 2.10 (s, 3H), 1.31 (s, 3H), 1.14 (s, 3H), 1.02 (s, 3H), 1.01 (s, 3H), 0.38 (s, 3H). ^13^C-NMR (DMSO-*d*_6_): δ 178.4, 170.5, 164.9, 148.7, 134.1, 127.3, 119.5, 118.0, 117.1, 77.2, 55.6, 55.4, 49.8, 46.7, 45.1, 43.0, 40.4, 39.3, 38.1, 36.3, 36.2, 35.0, 33.9, 33.5, 31.6, 31.2, 30.1, 29.4, 28.7, 25.0, 24.8, 21.8, 18.3, 10.3. HRMS (ESI) *m/z* calcd for C_37_ H_53_ N_2_ O_3_ [M + H]^+^: 573.4056, found: 573.4062. HPLC purity: 98.9%.

*(2R,4aS,6aS,12bR,14aS,14bR)-N-(2-((7-chloroquinolin-4-yl)amino)ethyl)-10-hydroxy-2,4a,6a,9,12b,14a-hexamethyl-11-oxo-1,2,3,4,4a,5,6,6a,11,12b,13,14,14a,14b-tetradecahydropicene-2-carboxamide* (**6**). Yield: 50%; red solid. Mp: 165.7–167.9 °C. ^1^H-NMR (DMSO-*d*_6_): δ 8.65 (s, 1H), 8.34 (d, *J* = 7.2 Hz, 1H), 8.22 (d, *J* = 8.7 Hz, 1H), 8.02 (bs, 1H), 7.83 (s, 1H), 7.54 (d, *J* = 8.7 Hz, 1H), 6.96 (d, *J* = 5.7 Hz, 1H), 6.60 (d, *J* = 7.2 Hz, 1H), 6.26 (s, 1H), 6.13 (d, *J* = 5.7 Hz, 1H), 3.36–3.25 (m, 4H), 2.10 (s, 3H), 1.31 (s, 3H), 1.14 (s, 3H), 1.02 (s, 3H), 1.01 (s, 3H), 0.38 (s, 3H). ^13^C-NMR (DMSO-*d*_6_): δ 179.3, 179.1, 178.5, 165.9, 151.1, 150.6, 146.2, 135.3, 134.4, 127.6, 127.4, 125.2, 122.6, 119.4, 118.2, 117.28, 93.4, 45.1, 44.5, 43.2, 40.1, 39.5, 38.4, 37.2, 36.3, 35.2, 33.5, 31.7, 31.4, 30.9, 30.5, 29.6, 28.8, 28.3, 21.8, 18.6, 10.5. HRMS (ESI) *m/z* calcd for C_40_H_48_ClN_2_O_3_ [M + H]^+^: 654.3462, found: 654.3463. HPLC purity: > 97.9%.

*(2R,4aS,6aS,12bR,14aS,14bR)-N-(3-((7-chloroquinolin-4-yl)amino)propyl)-10-hydroxy-2,4a,6a,9,12b,14a-hexamethyl-11-oxo-1,2,3,4,4a,5,6,6a,11,12b,13,14,14a,14b-tetradecahydropicene-2-carboxamide* (**7**). Yield: 59%; red solid. Mp: 211.1–215.0 °C. ^1^H-NMR (DMSO-*d*_6_): δ 8.71 (s, 1H), 8.33 (d, *J* = 5.4 Hz, 1H), 8.19 (d, *J* = 9.0 Hz, 1H), 7.75 (s, 1H), 7.64 (s, 1H), 7.41 (d, *J* = 8.7 Hz, 1H), 7.29 (s, 1H), 7.03 (d, *J* = 6.9 Hz, 1H), 6.37 (m, 2H), 1.74–1.55 (m, 12), 1.35 (s, 3H), 1.28–1.21 (m, 5H), 1.18 (s, 3H), 1.07 (s, 3H), 1.04 (s, 3H), 0.85–0.84 (m, 2H), 0.50 (s, 3H). ^13^C-NMR (DMSO-*d*_6_): δ 179.1, 179.0, 178.3, 165.8, 150.9, 150.4, 146.0, 135.1, 134.2, 127.5, 127.3, 125.2, 122.3, 119.3, 118.0, 117.27, 93.3, 45.0, 44.2, 43.0, 40.0, 39.3, 38.1, 37.1, 36.2, 34.9, 33.4, 31.5, 31.0, 30.7, 30.3, 30.0, 29.4, 28.6, 28.2, 21.7, 18.4, 10.3. HRMS (ESI) *m/z* calcd for C_41_H_50_ClN_2_O_3_ [M + H]^+^: 668.3613, found: 668.3616. HPLC purity: >99%.

*S-(((2R,4aS,6aS,12bR,14aS,14bR)-10-hydroxy-2,4a,6a,9,12b,14a-hexamethyl-11-oxo-1,2,3,4,4a,5,6,6a,11,12b,13,14,14a,14b-tetradecahydropicene-2-carboxamido)methyl) methanesulfonothioate* (**8**). Yield: 58%; red solid. Mp: 188.1–191.8 °C. ^1^H-NMR (CDCl_3_): δ 7.05 (d, *J* = 6.3 Hz, 1H), 6.57 (s, 1H), 6.37 (m, 2H), 3.52–3.44 (m, 2H), 3.34 (s, 3H), 3.26–3.22 (m, 2H), 2.45 (d, *J* = 15.6 Hz, 2H), 2.22 (s, 3H), 2.15–1.48 (m, 12H), 1.44 (s, 3H), 1.26 (s, 3H), 1.16 (s, 3H), 1.12 (s, 3H), 0.98–0.91 (m, 1H), 0.60 (s, 3H). ^13^C-NMR (CDCl_3_): δ 178.7, 164.9, 146.0, 134.5, 127.3, 119.4, 118.1, 117.4, 77.2, 65.8, 50.4, 45.1, 44.3, 43.1, 40.5, 39.4, 38.2, 36. 3, 35.4, 35.0, 33.5, 31.6, 31.0, 30.8, 30.0, 29.4, 28.6, 21.7, 18.5, 15.3, 10.3. HRMS (ESI) *m/z* calcd for C_32_H_46_NO_5_S_2_ [M + H]^+^: 610.2637, found: 610.2637. HPLC purity: >99%.

*S-(2-((2R,4aS,6aS,12bR,14aS,14bR)-10-hydroxy-2,4a,6a,9,12b,14a-hexamethyl-11-oxo-1,2,3,4,4a,5,6,6a,11,12b,13,14,14a,14b-tetradecahydropicene-2-carboxamido)ethyl) methanesulfonothioate* (**9**). Yield: 61%; orange solid. Mp: 178.4–181.9 °C. ^1^H-NMR (DMSO-*d*_6_): δ 8.66 (s, 1H), 7.64 (bs, 1H), 7.05 (d, *J* = 6.9 Hz, 1H), 6.39 (s, 1H), 6.33 (d, *J* = 7.2 Hz, 1H), 3.42 (s, 3H), 3.11 (t, *J* = 7.1 Hz, 2H), 2.96 (t, *J* = 7.1 Hz, 2H), 2.43 (d, *J* = 15.6 Hz, 2H), 2.09–2.06 (m, 2H), 2.07 (s, 3H), 2.01–1.47 (m, 12H), 1.37 (s, 3H), 1.20 (s, 3H), 1.06 (s, 3H), 1.03 (s, 3H), 0.98–0.91 (m, 1H), 0.51 (s, 3H). ^13^C-NMR (DMSO-*d*_6_): δ 178.4, 164.8, 146.0, 134.3, 127.4, 119.5, 118.1, 117.3, 77.2, 65.8, 50.5, 45.1, 44.3, 43.1, 40.4, 39.4, 38.2, 37.4, 36.3, 35.0, 33.8, 33.7, 33.5, 31.6, 31.0, 30.8, 30.0, 29.4, 29.4, 28.7, 18.4, 15.2. HRMS (ESI) *m/z* calcd for C_33_H_48_NO_5_S_2_ [M + H]^+^: 624.2793, found: 624.2802. HPLC purity: >98.6%.

*(2R,4aS,6aS,12bR,14aS,14bR)-N-(2-(allyldisulfanyl)ethyl)-10-hydroxy-2,4a,6a,9,12b,14a-hexamethyl-11-oxo-1,2,3,4,4a,5,6,6a,11,12b,13,14,14a,14b-tetradecahydropicene-2-carboxamide* (**10**). Yield: 53%; red solid. Mp: 173.7–175.1 °C. ^1^H-NMR (DMSO-*d*_6_): δ 8.65 (s, 1H), 7.78 (m, 1H), 7.06 (m, 1H), 6.35 (m, 2H), 5.85–5.76 (m, 1H), 5.10–5.04 (m, 2H), 3.20 (m, 2H), 2.69–2.64 (m, 4H), 2.07 (s, 3H), 1.79–1.52 (m, 13H), 1.36 (s, 3H), 1.19 (s, 3H), 1.05 (s, 3H), 1.02 (s, 3H), 0.83 (m, 1H), 0.52 (s, 3H). ^13^C-NMR (DMSO-*d*_6_): δ 178.3, 177.9, 170.3, 164.8, 146.0, 134.1, 133.2, 127.4, 119.5, 118.9, 118.0. 117.1, 45.0, 44.3, 42.0, 40.4, 39.4, 38.2, 38.0, 37.6, 36.4, 34.0, 33.8, 33.5, 31.6, 31.0, 30.9, 30.1, 29.5, 28.7, 21.7, 18.5, 10.3. HRMS (ESI) ***m/z*** calcd for C_34_H_48_NO_3_S_2_ [M + H]^+^: 587.4213, found: 587.4218. HPLC purity: >99%.

*(6bS,8aS,11R,12aR,12bS,14aR)-3-hydroxy-4,6b,8a,11,12b,14a-hexamethyl-11-(((((2R,3R,4R,5S,6R)-2,4,5-trihydroxy-6-(hydroxymethyl)tetrahydro-2H-pyran-3-yl)amino)oxy)carbonyl)-7,8,8a,9,10,11,12,12a,12b,13,14,14a-dodecahydropicen-2(6bH)-one* (**11**). Yield: 22%; red solid. Mp: 185.4–187.8 °C. ^1^H-NMR (acetone-d_6_): δ 7.54 (s, 1H), 7.13 (d, *J* = 6.3 Hz, 1H), 6.58 (bs, 1H), 6.44 (s, 1H), 6.42 (s, 1H), 5.66 (bd, 1H), 5.12 (s, 1H), 4.11 (bd, 1H), 3.88 (s, 1H), 3.76–3.69 (m, 3H), 3.63–3.55 (m, 1H), 3.42–3.27 (m, 2H), 2.76 (s, 1H), 2.53 (d, J = 15.3 Hz, 1H), 2.23 (s, 1H), 2.17 (s, 3H), 1.99–1.53 (m, 12H), 1.43 (s, 3H), 1.29 (s, 3H), 1.21 (s, 3H), 1.14 (s, 3H), 0.98–0.91 (m, 1H), 0.78 (s, 3H). ^13^C-NMR (acetone -*d*_6_): δ 178.3, 177.6, 177.1, 169.5, 133.6, 127.2, 119.5, 118.0, 116.6, 91.4, 72.03, 71.9, 71.9, 62.5, 62.0, 54.7, 44.9, 42.6, 40.0, 39.3, 37.7, 36.4, 35.0, 33.4, 33.0, 31.1, 31.0, 29.9, 21.3, 21.2, 18.5, 9.4. HRMS (ESI) *m*/*z* calcd for C_35_H_50_NO_8_ [M + H]^+^: 634.3359, found: 634.3356. HPLC purity: 98.9%.

**HPLC Analysis**

Instrument: VWR Hitachi system equipped with a L2130 Pump, L-2450 Diode Array Detector, L-2300 Column Oven and L-2200Autosampler. Column: LIChroCART 250–4 RP-18e (5 µm). Temperature: 45 °C.

Gradient:

- Phase A: H_2_O/CH_3_CN/TFA 97:3:0.1
- Phase B: H_2_O/CH_3_CN/TFA 30:70:0.1

**Table A1:** HPLC gradinet detalis.

Time (min)	% A	% B	Flux (mL/min)
0	100	0	0
1	100	0	1
18	0	100	1
22	0	100	1
24	0	100	1
30	100	0	1
31	100	0	0

## Appendix B

***Lb*Hsp90 full sequence:**

>XP_001567804.1 heat shock protein 83–1 [*Leishmania braziliensis* MHOM/BR/75/M2904]

MTETFAFQAEINQLMSLIINTFYSNKEIFLRELISNASDACDKIRYQSLDPSVLGEE- THLRVRVVPDKA

NKTLTVEDNGIGMTKADLVNNLGTIARSGTKAFMEALEAGGDMMIGQFGVGF- YSAYLVADRVTVVSKNN

SDEAYVWESSAGGTFTITSVPESDMKRGTRITLHLKEDQQEYLEERRVKELIKKHS- EFIGYDIELMVEKT

AEKEVTDEDEEEDESKKKSCGDEGEPKVEEVTEGGEDKKKKTKKVKETTYEVQN KHKPLWTRDPKDVT

KEEYAAFYKAISNDWEDPAATKHFSVEGQLEFRAIAFVPKRAPFDMFENKKRNNIKLYVRRVFIMDNCE

DLCPDWLGFVKGVVDSEDLPLNISRENLQQNKILKVIRKNIKKCLELFEEIAENK- EDYKQFYEQFGKNI

KLGIHEDTANRKKLMELLRFYSTESGEEMTTLKDYVTRMKPEQKSIYITGDSKKKLESSPFIEKARRCG

LEVLFMTEPIDEYVMQQVKDFEDKKFACLTKEGVHFEESEEEKKQREEKAACEK- LCKTMKEVLGDKVEK

VTVSERLSTSPCILVTSEFGWSAHMEQIMRNQALRDSMAQYMVSKKTMEVNPD-HPIIKELRRRVEADEN

DKAVKDLVFLLFDTSLLTSGFQLDDPTGYAERINRMIKLGLSLDEEDEEVAEAPPAEAAPAEVTAGTSSMEQVD

*Lb*Hsp90 inhibition (%) = (1 − *k*/*k*_native_) * 100          *k = ΔAbs/min = * extrapolated apparent kinetic constant (Abs/min)

**Table A2:** **CE**, compound **3** and **GA** *Lb*Hsp90 ATPase kinetics modulation at 50 μM.

Compounds (50 μM)	*Lb*Hsp90 Inhibition (%) ^1^
**CE**	28.9 ± 5.4
**3**	97.8 ± 1.2
**GA**	<0.05

^1^ All compounds were tested two times in triplicate and reported data are the corresponding arithmetic mean.

**Table A3:** **GA** dose-response data for *Lb*Hsp90 ATPase kinetics modulation.

GA Concentration (μM)	*Lb*Hsp90 Inhibition (%) ^1^
10	0.1 ± 0.02
15	0.2 ± 0.05
20	0.3 ± 0.08
50	0.7 ± 0.04
100	6.5 ± 0.9
200	30.7 ± 1.2
400	52.3 ± 0.8
600	76.9 ± 3.0
800	90.5 ± 2.5
1000	99 ± 0.9
1200	>99

^1^ Tests were two times in triplicate and reported data are the corresponding arithmetic mean.
